# Biphasic Production of Anti-ApoB100 Autoantibodies in Obese Humans and Mice

**DOI:** 10.3390/ph14040330

**Published:** 2021-04-04

**Authors:** Moon Kyung Choe, Hyung-Ji Kim, Nan Hee Kim, Bert Binas, Hyo Joon Kim

**Affiliations:** 1 Department of Molecular & Life Science, College of Science & Technology, Hanyang University (ERICA), 55 Hanyangdaehak-ro, Sangnok-gu, Ansan, Gyeonggi-do 15588, Korea; jjys71@threehbio.com; 2 Asan Medical Center, Department of Neurology, University of Ulsan College of Medicine, 88, Olympic-ro 43-gil, Songpa-gu, Seoul 05505, Korea; garailsikzip@gmail.com; 3 Department of Internal Medicine, Division of Endocrinology and Metabolism, College of Medicine, Korea University, 145 Anam-ro, Seongbuk-gu, Seoul 02841, Korea; nhkendo@gmail.com

**Keywords:** obese patients, apolipoprotein B-100, IgG-type autoantibody, high-fat diet induced obesity, epitope, mimotope, body mass index

## Abstract

Obesity is associated with autoimmunity, a phenomenon considered as harmful. Here we show that obese mice and humans produce IgG-type autoantibodies that specifically recognize apolipoprotein B-100 (ApoB100), its native epitope p210, and the synthetic p210 mimotope pB1. By contrast, antibodies against epitopes p45 and p240, which have been associated with atherosclerosis, were not detected in either the humans or mice. In a longitudinal analysis of high fat diet-fed mice, autoantibody production rose with increasing body weight, then decreased and plateaued at morbid obesity. Likewise, in a cross-sectional analysis of sera from 148 human volunteers spanning a wide BMI range and free of comorbidities, the immunoreactivity increased and then decreased with increasing BMI. Thus, the obesity-related ApoB100-specific natural autoantibodies characteristically showed the same epitope recognition, IgG-type, and biphasic serum levels in humans and mice. We previously reported that a pB1-based vaccine induces similar antibodies and can prevent obesity in mice. Therefore, our present results suggest that autoantibodies directed against native ApoB100 may mitigate obesity, and that the vaccination approach may be effective in humans.

## 1. Introduction

Many countries are experiencing an obesity epidemic, for which an effective therapy has not yet been found [1,2,3,4]. An intriguing facet of obesity is its association with autoimmunity and related comorbidities involving adipokines and cytokines [5,6]. A range of autoimmune antibodies has been found to be produced in obesity [7,8], but little is known about their roles. Generally, autoantibodies are seen as components that aggravate pathogenesis [9,10]. However, studies of rheumatoid arthritis, atherosclerosis, type 1 diabetes, and Sjögren’s syndrome have raised the possibility that certain autoantibodies may have beneficial, protective effects [11,12,13,14,15,16,17]. For example, it was found that autoantibodies against p210, a native epitope of human ApoB100, are associated with a reduced severity of atherosclerosis in humans [18,19], and immunization of mice with antibodies directed against native p210 had an anti-atherogenic effect [20,21], raising the possibility of a similar role of the corresponding autoantibodies. We previously found that pB1, a synthetic mimotope of p210, can induce antibodies that recognize native p210 and ApoB100 and prevent diet-induced obesity in mice [22]. We therefore speculate that in analogy with the findings on atherosclerosis, certain ApoB100/p210-reactive autoantibodies may be part of an anti-obesity, rather than pathogenic, mechanism. Here, we show that obese mice and humans produce autoantibodies that recognize ApoB100/p210/pB1. Remarkably, the antibody titers do not simply increase with the body weight but first increase and then stop increasing, and even decrease again.

## 2. Results

### 2.1. Metabolic Context

This study analyzed sera from wild-type chow- or high-fat diet (HFD)-fed mice and from humans of a wide BMI range. We first obtained profiles of lipids and liver enzymes. In both species, the serum triglyceride levels did not change significantly with higher weights (Supplementary Figure S1A,B). In contrast, the cholesterol values (total, HDL, LDL) increased in mice but not humans; we note, however that comparability is limited by the fact that the mouse study was longitudinal, while the human study was cross-sectional. Nevertheless, in both humans and mice the body weight increases ultimately led to moderately increased serum levels of AST or ALT (Supplementary Figure S1A,B). More importantly, since diabetic and cardiovascular patients were excluded from the study (see Section 4.2), it should be noted that the obese mice were insulin-resistant but not diabetic (Supplementary Figure S1A) and that wild type mice do not develop atherosclerosis [23,24]. Thus, the two rather different species were physiologically comparable as much as is possible.

### 2.2. Obese Mice and Humans Produce ApoB100/p210/pB1-Specific Autoantibodies

**Figure 1:**
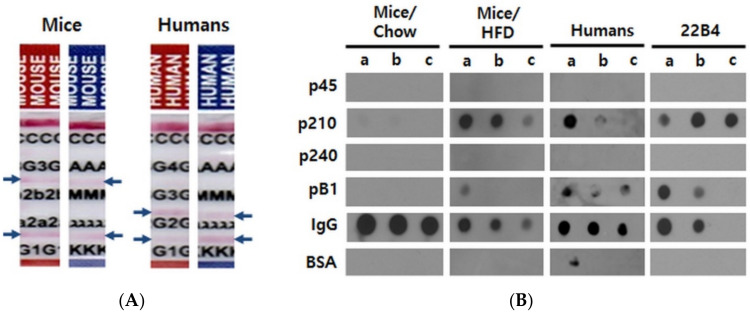
Characterization of autoantibodies in mouse and human sera. (**A**) Determination of isotypes. Antibodies were affinity-purified from pooled sera over human ApoB100 and analyzed with paper strip isotyping kits. Blue arrows indicate positive reactions. C; control, G3; IgG3, 2b; IgG2b, 2a; IgG2a, G1; IgG1, G4; IgG4, G2; IgG2, A; IgA, M; IgM, λ; lambda light chain, κ; kappa light chain. (**B**) Epitope mapping of ApoB100 affinity-purified mouse and human antibodies as well as of mouse monoclonal antibody 22B4 raised against pB1. The mouse sera used for affinity purification were harvested and pooled at the end of the experiments (33 weeks of age) from Chow-fed or HFD-fed mice. The human sera were pooled after the first ELISA screening experiments and also affinity-purified before the dot blot analysis. 0.2 µg of the given antibody preparation were added per well. a, b and c indicate the amounts of the peptides or proteins spotted per well; a, b and c = 5, 2.5 and 1 µg for p45, p210, p240, pB1 and BSA (bovine serum albumin; negative control); a, b and c = 5, 2.5 and 1 ng for immunoglobulins (M/H IgG = mouse or human IgG; positive control), respectively.

In order to characterize the ApoB100-specific autoantibodies from human and mouse, we separately pooled the sera of 16 obese mice (after 15 weeks of HFD) and of 148 human subjects. We loaded the pooled mouse or human sera on Affi-gel linked ApoB100 affinity chromatography columns and in both cases were able to elute an immunoglobulin fraction, indicating the existence of ApoB100-specific autoantibodies. We determined their isotypes as IgG1, IgG2b, and IgM with kappa light chains in the mice, and as IgG1 and IgG2 type with kappa and lambda light chains in the human subjects (Figure 1A).

We then performed dot blot experiments in order to clarify whether these antibodies would recognize the previously known human ApoB100 autoantibody epitopes, p45, p210, and p240; these peptides are homologous to their mouse counterparts by 95%, 90%, and 60%, respectively (UniProtKB/Swiss-Prot accession no. P04114.2 for human and E9Q414.1 for mouse). Both the human and mouse affinity-purified antibodies reacted with pB1 and with p210, of which pB1 is a mimotope. Unexpectedly, however, no reactivity was found with p45 and p240 (Figure 1B). Importantly, the exact same reactivity pattern was exhibited by the monoclonal antibody 22B4 (Figure 1B) that we had previously raised against pB1 [22]. Thus, sera from obese mice and humans contain autoantibodies directed against the ApoB100 epitope p210, and these autoantibodies also recognize the synthetic p210 mimotope pB1.

### 2.3. The Autoantibody Response in Obese Mice Exhibits a Biphasic Pattern

**Figure 2:**
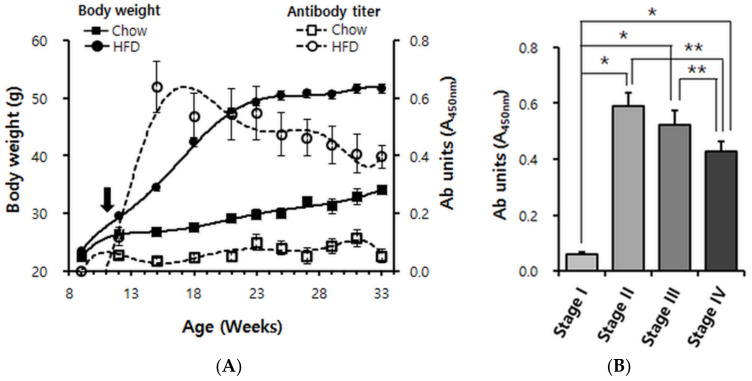
Longitudinal analysis of anti-pB1 antibody titers and body weight in Chow-fed and high fat diet (HFD)-fed mice. (**A**) Body weight curves and superimposition of autoantibody titer. Male C57BL/6 mice were continuously maintained on Chow (*n* = 11; squares) or switched (black arrow) at 11 weeks of age to a 60% HFD (*n* = 16; circles). Solid symbols indicate body weights; empty symbols indicate antibody units (absorbance of ×100 diluted serum at 450 nm). Data points are shown for every 3rd week, i.e., 9, 12, 15, 18, 21, 24, 27, 30 and 33 weeks of age. (**B**) Cluster analysis of the data shown in A. Based on the degree of obesity, its evolution was divided into four stages. Stage I: 9~12 weeks of age (overweight), stage II: 13~18 weeks of age (mildly obese), stage III: 19–25 weeks of age (moderately obese), stage IV: 26–33 weeks of age (severely obese). Error bars in panels A and B indicate means ± s.e.m. * *p* < 0.0001, ** *p* < 0.01.

Next, we performed ELISA measurements in order to characterize the anti-ApoB100/p210 autoimmune response throughout the evolution of obesity in mice fed a HFD; the autoantibody characterization outlined in the foregoing section allowed us to use pB4, which is more practical than ApoB100 or p210, as the diagnostic antigen. At 11 weeks of age, a group of male mice that had been kept on a regular chow was split into two: one group continued to receive the chow, the other one was switched to HFD. Starting from week 9 and up to 33 weeks of age, every three weeks, we measured the body weights and antibody titers for the ApoB100 mimotope pB1. The chow-fed mice showed a continuous but modest increase in body weight, reaching ~33 g between 30 and 33 weeks of age. These mice remained lean [25], and no pB1-reactive antibodies were detected in their sera during the whole experimental period (Figure 2A).

In contrast, the HFD-fed mice became highly obese. They achieved most of their weight gain in a steep rise by 21 weeks of age and reached their final weight of ~50 g between 24 and 27 weeks of age. Already by 12 weeks of age (a week after the switch to HFD), when the HFD-fed mice were only ~3 g heavier than the chow-fed mice, an immunoreactivity against pB1 was clearly detectable (Figure 2A). The titer increased steeply until ~15 weeks of age (4 weeks after the switch to HFD). Unexpectedly, the titer then declined again but nevertheless remained high till the end of the experiment, where it appeared to reach a plateau (Figure 2A). Using the Friedman test algorithm, we divided the evolution of HFD-induced obesity into four stages. At stage I (week ages between 9–12, overweight) antibody induction was detectable; at stage II (week ages between 13–18, mildly obese) antibody levels increased dramatically; at stage III (week ages between 19–25, moderately obese) antibody levels did not increase further but appeared to decline (not significant); but at stage IV (week ages after 26, severely obese) antibody levels clearly declined compared to stages II and III (Figure 2B). This provided the clearest illustration of the rise and subsequent decline of the anti-ApoB100/p210 autoimmune response.

### 2.4. The Autoantibody Response in Obese Humans Also Exhibits a Biphasic Pattern

**Figure 3:**
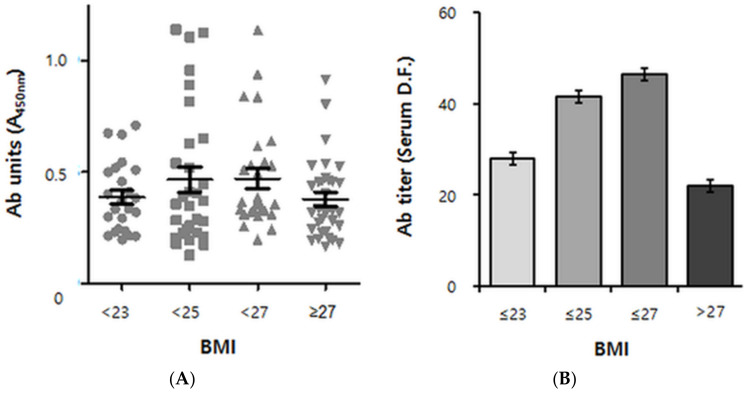
Cross-sectional analysis of human anti-pB1 antibody titer data versus BMI. (**A**) Scatterplot derived from 107 positive samples (out of 148 human volunteers of South Korean nationality) according to the BMI group. BMI < 23: lean, 23 ≤ BMI < 25: overweight, 25 ≤ BMI < 27: obese, 27 ≤ BMI: severely obese. *n* = 23, 26, 27 and 31, respectively. Ab (antibody) units indicate the absorbance of ×100 diluted sera at 450 nm. Error bars indicate means ± s.e.m. (**B**) Quadratic Trend Analysis of data shown in A. Antibody titers refer to serum dilution folds (D.F.) yielding an absorbance read-out of 0.5 (See “Supplementary Materials” for a detailed explanation). The association between autoantibody titers and the BMI group was adjusted for age and sex. The *P* value for quadratic trend was 0.0494 (*p* < 0.05). Error bars indicate geometric means ± s.e.m.

In humans, a longitudinal analysis analogous to that done in mice is not practical. We therefore performed a cross-sectional study of ApoB100 immunoreactivity for BMI values ranging from ~20 (lean) to ~36 (severely obese). Although immunoreactivity against ApoB100 was unambiguously detected in a first screening step (see Section 4.4), there was significant variation, and a robust pattern was not readily apparent from the scatter plot (Supplementary Figure S2A). We therefore performed a second screening step designed to eliminate false positive samples, using the fact that the ELISA absorbance read-outs of truly positive samples should linearly decrease with increased dilution (Supplementary Figure S2B). Based on the dot blot results (Figure 1), we were able to replace ApoB100 by pB4 as analyte in the 2nd screening step. Out of the 148 samples used in the first ELISA step (Supplementary Figure S2A), 107 samples passed the second screening (Figure 3A). After these data were adjusted for sex and age, quadratic trend analysis was performed (Figure 3B).

As a result, a biphasic pattern emerged, with the antibody titers ascending at BMIs up to 27 kg/m^2^ but descending thereafter, resulting in an inverted U-shape (P for quadratic trend of 0.0494) (Figure 3B). Remarkably, the autoantibody positive/negative populations showed the biphasic pattern as well (Supplementary Figure S3).

## 3. Discussion

Since we have previously shown that a pB1-based vaccine (which induced antibodies that reacted with p210 and ApoB100) can prevent HFD-induced obesity in mice [22], our new finding suggests that obesity-induced anti-p210 autoantibodies may blunt the weight increase in non-vaccinated mice. In this respect, it is intriguing that in both mice and humans, the antibody titers did not continuously rise with the degree of obesity but rather decreased and leveled off in morbid obesity. Such a biphasic pattern seems consistent with the hypothesis that the autoimmune antibody mechanism is protective but becomes overwhelmed at morbid obesity.

Our findings resemble previous findings in atherosclerosis, where p210 is also targeted by autoantibodies [18,19], and vaccination with that antigen counteracts atherogenesis [21,26,27]. How different these two contexts really are, however, awaits further study: Obesity and atherosclerosis occur frequently (but not necessarily) together [28,29], hence there may be mechanistic overlap (one possibility is outlined in the next paragraph). On the other hand, in our study the obesity-associated autoantibodies did not recognize two other ApoB100 epitopes, p45 and p240, which (in addition to p210) were recognized by autoantibodies in atherosclerosis [30,31,32]. It should be noted, however, that the kinetics of the two diseases are different. Obesity develops much more rapidly, which could explain why we did not observe an effect of transient anti-obesity vaccination with pB1 on atherogenesis in ApoE knockout mice [33].

Further work is required to elucidate the mechanisms of obesity-associated anti-p210 autoantibody induction. It was previously shown that in obesity, the macrophage-derived protein, AIM, boosts the production of multiple IgG autoantibodies by promoting the presentation of IgM-bound antigens to B cells [7]. In this mechanism, a given IgG epitope must be located on the same antigen as one of the IgM epitopes. It is therefore of interest that in atherosclerosis—another inflammatory context involving macrophages—ApoB100 fragments of 15 kDa and 42 kDa in size were observed that carry the IgM epitope p216 [30,34], which corresponds to amino acids 3226–3245 of ApoB100. Since this is close to p210 (aa 3136–3155 of ApoB100), it appears plausible that both epitopes are present on the same ApoB100 fragment and that hence the IgG-type anti-p210-autoantibodies described in the present report were also induced via the AIM/p216 mechanism. Note that once the plasma cells triggered by this non-classical mechanism become exhausted, the corresponding memory B cells will hardly become activated because they lack the support of T helper cells (which are eliminated by the self-tolerance mechanisms) and will outcompete the natural autoantibodies (which are of IgM type and therefore of lower affinity)—potentially explaining why the autoimmune response is not further increased or even decreased in morbid obesity.

In conclusion, we show here that in both mice and humans, obesity is associated with the production of autoantibodies that are directed against p210, an epitope of ApoB100, and can also recognize the synthetic p210 mimotope pB1. Moreover, in both species the antibody titers appear to go through a peak before reaching the morbid stage, or at least do not further increase in morbid obesity. In conjunction with our previous study demonstrating an obesity-preventing effect of a pB1-based vaccine [22], these findings (i) add obesity to the range of pathologies in which autoantibodies potentially play a protective physiological role (see Introduction) and (ii) provide support for the rationale [22] that the anti-obesity vaccination strategy that was successful in mice can be extended into humans.

## 4. Materials and Methods

### 4.1. Animals, Diets, and Serum Harvest

Seven weeks old male C57BL/6 mice were purchased from SLC, Inc. (Seoul, Korea). The animals were kept in a temperature- and light-controlled room (25 °C, 12 h light and 12 h dark cycle) and allowed free access to water and food. From 11 weeks (w) of age, the animals were given either a low-fat diet (10 kcal% fat by calories, #D1250B, “Chow”) or a high-fat diet (60 kcal% fat by calories, #D12492, “HFD”) from Research Diets, Inc. (New Brunswick, NJ, USA). Each group included 11 chow-fed and 16 HFD-fed mice. The mice were weighed three times per week, and the average weight was determined for each week. Blood was taken by eye-bleeding every 3rd week from 9 till 33 weeks of age. For preparation of serum, the blood was drawn into a tube, left at room temperature for 30 min, and then centrifuged (13,000 rpm, 10 min, 4 °C). Biochemical analysis of the lipid profiles was performed at weeks 9 (before the HFD), 23, and 33 (time of sacrifice) of age (Supplementary Figure S1). All animal procedures were approved by the Center for Laboratory Animal Science of Hanyang University and the Institutional Review Board (HY-IACUC-19-0048).

### 4.2. Human Subjects and Serum Harvest

The serum samples used in this study had been prepared in 2015–2016 [35] and stored at −80 °C until use. 148 volunteer participants (80 males, 68 females) between 30 and 70 years of age were recruited during routine health checks at the Center for Health Promotion at Korea University Ansan Hospital. All participants responded to an interviewer-administered questionnaire and underwent a comprehensive physical examination. Lifestyle characteristics included smoking status and alcohol consumption categorized as never, former, and current. Subjects with a history of chronic illness, including diabetes mellitus, hypertension, or cardiovascular disease were excluded. Blood was drawn for biochemical analysis by using serum separator tubes (Vacutainer; Becton Dickinson, Franklin Lakes, NJ, USA) after an overnight fast. The participants signed an informed consent form for usage of stored samples for other studies. The baseline clinical characteristics of the study participants are shown in Table 1. This study was performed according to the principles of the Declaration of Helsinki of the World Medical Association and was approved by the Institutional Review Committee at Korea University Ansan Hospital (AS17011).

### 4.3. Peptide Preparation

The previously defined [30] peptides p45 (IEIGLEGKGFEPTLEALFGK), p210 (KTTKQSFDLSVKAQYKKNKH), and p240 (FPDLGQEVALNANTKNQKIR), as well as peptide pB1 (RNVPPIFNDVYWIAF) were chemically synthesized by Peptron Co. (Daejeon, Korea). Peptide pB4, a tandem 4-repeat of pB1, was expressed in *E. coli* and purified as previously described [22].

### 4.4. Serum Antibody Analysis

**Figure 4:**
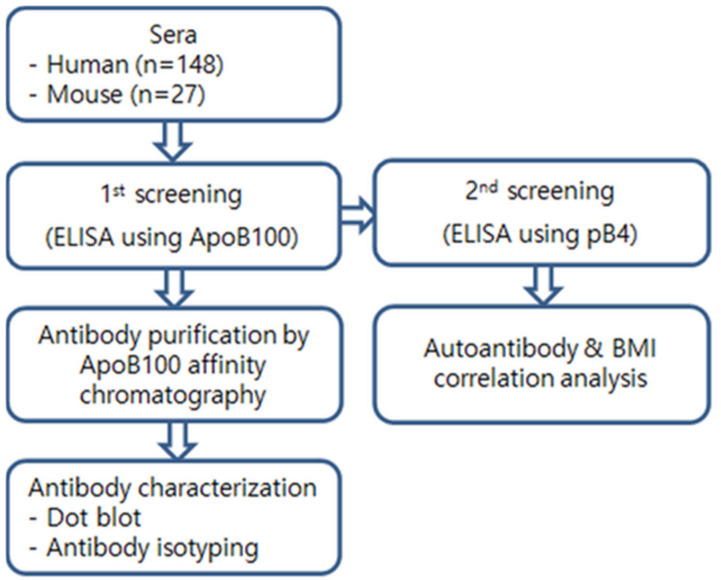
Strategy of serum analyses.

Figure 4 gives an overview of the usages of the human and mouse sera. All the serum samples were heat-inactivated at 56 °C for 30 min to destroy complement factors. Indirect ELISA was performed in 96-well plates (#32296, SPL Life Science, Pocheon, Korea) with a standard protocol. Each well bottom was coated with 100 ng of ApoB100 (for the 1st screening) or pB4 (for the 2nd screening) in an overnight incubation at 4 °C, then washed with PBS-T (PBS with 0.05% Tween-20) and blocked with a 0.05% casein solution for 2 h at 37 °C. The wells were then incubated for 1 h at 37 °C with 100 µL of diluted mouse or human serum, washed, and then incubated for 1 h at 37 °C with HRP-conjugated anti-mouse IgG (0.1 µg/mL, A0168, Sigma, St. Louis, MO, USA) or HRP-conjugated goat anti-human IgG, IgM, IgA (H + L) (#31418, Invitrogen, Carlsbad, CA, USA), respectively.

The color reaction was performed with *o*-phenylenediamine dihydrochloride for 10 min at 37 °C, and the absorbency (450 nm) measured to calculate the antibody titer. Human ApoB100 was purchased from Sigma (A5353, Sigma). Because of high background values especially of the human sera, we used a two-stage ELISA screening procedure (Figure 4) in order to discriminate true from false positive signals. In the first screening stage, we used 100-fold diluted sera with human ApoB100 as analyte (Supplementary Figure S2A). In the second screening step, the sera were serially diluted 100-, 200-, 400-, and 800-fold and pB4 used as analyte. Samples were considered as positive when they showed a first-order linear relationship between dilution fold and absorbance with a negative slope X > 0.074 (See “Supplementary Materials” for a detailed explanation).

### 4.5. Purification of Autoantibodies against ApoB100

Sera were separately pooled from 16 HFD-fed mice (collected from weeks 15 to 33, Figure 2) or from 148 humans. The antibodies were purified from these sera by affinity chromatography using human ApoB100-conjugated Affi-gel 15 resin. Conjugation of ligand and resin was conducted according to the instruction provided by the manufacturer with minor modifications. In brief, human ApoB100 (A5353, Sigma, St. Louis, MO, USA) was conjugated with 3 mg of freshly washed Affi-gel 15 (#153-6051, BIO-RAD, Hercules, CA, USA) gel in 0.1 M HEPES buffer (pH 7.5). The Affi-gel was mixed with 3 mL of ApoB100 (1 mg/mL) and the suspension was gently agitated on a wheel (10 rpm, 4 h at 4 °C). The coupling reaction was stopped by adding 0.3 mL of 0.1 M ethanolamine-HCl (pH 8.0) for 1 h and washed with PBS. The conjugated resin was then packed into an open column (φ1.0 cm × 3 cm), and 5 mL-aliquots of pooled anti-serum were applied to the top of the resin for three times followed by gravity-flow elution. Unbound or non-specific serum proteins were washed off (0.1 M sodium phosphate with 1 M NaCl, pH 7.4) prior to elution, and the ApoB100 specific antibodies were eluted with 0.1 M Glycine-HCl (pH 2.5). Eluates were neutralized with 2 M Tris-HCl (pH 7.5) and concentrated using VIVASPIN 500 (#VS0101, Sartorius, Göttingen, Germany). Finally, the eluates were dialyzed (molecular weight cut off 3000 Da) against 1 L of PBS overnight at 4 °C, and the VIVASPIN concentration was repeated for three cycles. The antibodies from chow-fed mice (11 mice, weeks 15 to 33) were purified using protein A (20333, Pierce, Waltham, MA, USA) rather than by ApoB100-affinity chromatography. 22B4 is a monoclonal antibody raised in-house against pB4 [22].

### 4.6. Dot-Blots

Synthetic peptides and proteins were dissolved in 10% DMSO at 1 mg/mL and diluted in PBS. A 0.2 μm PVDF membrane (#1620177, Bio-Rad, Hercules, CA, USA) was pre-wetted with methanol, washed with distilled water and then assembled into a 96-well Bio-Dot microfiltration apparatus (#1706545, Bio-Rad). Peptide or protein solutions were added to each well and allowed to filter through onto an absorption pad for 30 min. For immunoblotting, the membrane was blocked for 3 h at room temperature in 5% (*w*/*v*) non-fat dried skimmed milk in TBST (Tris-buffered saline with 0.1% Tween-20) and incubated overnight at 4 °C with the ApoB100 affinity purified antibody (0.2 µg protein/well, diluted with TBST). The membrane was then washed with TBST three times and incubated for 1 h at room temperature with HRP-conjugated goat anti-mouse IgG (Fc) (A0168, Sigma) or HRP-conjugated goat anti-human IgG/IgM/IgA (H + L) (#31418, Invitrogen), then washed three times with TBST, and finally developed using the ECL prime Reagent (RPN2232, GE Healthcare, Chicago, IL, USA).

### 4.7. Identification of Isotype

Antibodies were analyzed using the Iso-Gold rapid mouse or human antibody isotyping kit (mouse #MISOT-010, human #HISOT-010, BioAssay Works LLC, Ijamsville, MD, USA).

### 4.8. Data Analysis and Statistics

In the mouse experiments, the longitudinal period was divided into 4 stages based on antibody titer changes. The stages were denoted as: Stage I, overweight (week ages of 9~12); Stage II, mildly obese (week ages of 13~18); Stage III, moderately obese (week ages of 19~25); Stage IV, severely obese (week ages of 26~33). As antibody titer difference values violate the normality (Shapiro–Wilk test; *p* = 0.012 for Stage I vs. II, *p* = 0.0062 for Stage I vs. III, *p* = 0.0011 for Stage I vs. IV) and sphericity (*p* < 0.001) assumptions, the Friedman test with Wilcoxon signed-rank test as post-hoc analysis were used to evaluate repeated measures. For the human data, the antibody titers did not meet the normal distribution (Shapiro-Wilk test, *p* < 0.001). Therefore, the antibody titers were converted to their natural logarithms, and the effect of different BMI levels on the antibody titers was evaluated by Quadratic Trend Analysis with the contrast statement of PROC GLM (general linear model) of the SAS 9.4 program (SAS Institute Inc., Cary, NC, USA). The level of significance was set to *p* < 0.05.

## Figures and Tables

**Table 1:** Baseline characteristics of the human subjects by BMI.

Characteristics	BMI (kg/m^2^)			
	<23.0 (*n* = 33)	23.0 to <25.0 (*n* = 41)	25.0 to <27.0 (*n* = 30)	≥27.0 (*n* = 44)
Age (years)	54.4 ± 1.6	54.4 ± 1.5	54.3 ± 1.6	52.0 ± 1.4
Hight (cm)	162.2 ± 1.8	165.5 ± 1.2	163.2 ± 1.6	164.4 ± 1.5
Weight (kg)	58.0 ± 1.3	65.2 ± 1.0	69.7 ± 1.4	78.6 ± 1.5
BMI (kg/m^2^)	21.8 ± 0.1	23.8 ± 0.1	26.0 ± 0.1	29.2 ± 0.3
Diabetes (%)	0 (0.0)	0 (0.0)	0 (0.0)	0 (0.0)
Hypertension (%)	9 (27.3)	7 (16.7)	3 (10.0)	13 (39.4)
Dyslipidemia (%)	2 (6.1)	5 (11.9)	2 (6.7)	6 (18.2)
Smoking (%)	7 (21.2)	8 (19.0)	4 (13.3)	8 (24.2)
Alcohol (%)	12 (36.4)	21 (50.0)	14 (46.7)	22 (66.7)
Exercise (%)	14 (42.4)	22 (52.4)	15 (50.0)	19 (57.6)
WC (cm)	78.7 ± 1.1	83.8 ± 0.7	86.2 ± 0.6	92.3 ± 0.9
SBP (mm/Hg)	116.3 ± 2.3	120.8 ± 2.2	120.0 ± 2.3	121.7 ± 1.7
FPG (mg/dL)	97.1 ± 2.3	95.0 ± 1.9	92.4 ± 2.0	97.6 ± 1.6
HbA1c (%)	5.5 ± 0.1	5.4 ± 0.1	5.4 ± 0.1	5.5 ± 0.1
Total cholesterol (mg/dL)	201.9 ± 5.9	203.0 ± 5.9	194.8 ± 5.2	200.3 ± 5.5
Triglyceride (mg/dL)	141.6 ± 23.2	172.1 ± 14.5	142.5 ± 14.7	173.5 ± 15.2
LDL-C (mg/dL)	125.4 ± 5.0	117.0 ± 6.1	123.8 ± 4.9	122.3 ± 5.2
HDL-C (mg/dL)	48.5 ± 2.6	45.8 ± 1.8	47.6 ± 2.1	47.1 ± 1.8
hsCRP (mg/L)	0.5 ± 0.2	0.5 ± 0.1	2.5 ± 1.6	0.6 ± 0.1
AST (IU/L)	24.1 ± 1.2	24.2 ± 0.9	24.8 ± 1.2	26.9 ± 1.3
ALT (IU/L)	19.6 ± 2.2	21.4 ± 1.3	22.3 ± 1.5	32.6 ± 2.8
WBC (×103 cells/ul)	5.5 ± 0.2	6.2 ± 0.2	5.8 ± 0.2	6.2 ± 0.2

The human donors were categorized as BMI < 23 (lean), 23 ≤ BMI < 25 (overweight), 25 ≤ BMI < 27 (obese), or BMI ≥ 27 (severely obese) based on the Asian and Pacific Island population BMI standard. Data are presented as the mean ± s.e.m. Abbreviations: BMI, body mass index; WC, waist circumference; SBP, systolic blood pressure; FPG, fasting plasma glucose; LDL-C, low-density lipoprotein cholesterol; HDL-C, high-density lipoprotein cholesterol; hsCRP, high-sensitivity C-reactive protein; AST, aspartate aminotransferase; ALT, alanine aminotransferase; WBC, white blood cell.

## Data Availability

Data available in a publicly accessible repository.

## Supplementary Materials

The following Supplementary Method and Figures are available online at https://www.mdpi.com/article/10.3390/ph14040330/s1; Supplementary Figure S1: Serum analysis in mouse and human, Supplementary Figure S2: Establishing criteria for true autoantibody positivity, Supplementary Figure S3: Pattern of autoantibody extinction along obesity progression.
