# Supplementary material for: Biphasic Production of Anti-ApoB100 Autoantibodies in Obese Humans and Mice
Source: Pharmaceuticals (Basel). 2021 Apr 4;14(4):330. doi: 10.3390/ph14040330 (PMC8065440; doi:10.3390/ph14040330)
Supplement: Supplementary file 1 [file pharmaceuticals-14-00330-s001.pdf]

Supplementary Materials

Biphasic production of anti-ApoB100 autoantibodies in obese humans and mice

Moon Kyung Choe[1], Hyung-Ji Kim[2], Nan Hee Kim[3], Bert Binas[1] and Hyo Joon Kim[1]

[1] Department of Molecular & Life Science, College of Science & Technology, Hanyang University (ERICA), 55 Hanyangdaehak-ro, Sangnok-gu, Ansan, Gyeonggido, 15588, Republic of Korea; jjys71@threehbio.com
[2] Department of Neurology, University of Ulsan College of Medicine, Asan Medical Center, 88, Olympic-ro 43-gil, Songpa-gu, Seoul, 05505, Republic of Korea; garailsikzip@gmail.com
[3] Division of Endocrinology and Metabolism, Department of Internal Medicine, College of Medicine, Korea University, 145 Anam-ro, Seongbuk-gu, Seoul, 02841, Republic of Korea; nhkendo@gmail.com

Correspondence

Hyo Joon Kim, Department of Molecular & Life Science, College of Science & Technology, Hanyang University (ERICA) 55 Hanyangdaehak-ro, Sangnok-gu, Ansan, Gyeonggi-do, 15588, Republic of Korea

Tel: +82-10-9335-0001

E-mail: kimhj104@hanyang.ac.kr

Bert Binas, Department of Molecular & Life Science, College of Science & Technology, Hanyang University (ERICA) 55 Hanyangdaehak-ro, Sangnok-gu, Ansan, Gyeonggi-do, 15588, Republic of Korea

Tel: +82-10-5432-5517

E-mail: bbinas@hanyang.ac.kr

Supplementary methods

Discrimination of autoantibody-positive from negative human sera.

Positive and negative signals (including the false-positive read-outs caused by background signals) from human sera were discriminated by a second ELISA screening step using pB4 instead of ApoB100 as antigen (see Figure 4). This approach (i.e., changing the antigen to pB4) is based on the finding that the obesity-related p210-reactive autoantibodies simultaneously recognized pB4 (Figure 1B).

1) The second ELISA analysis was performed using serially (x100, x200, x400, and x800) diluted sera. From the plots of the dilution folds (D) versus the absorbance read-outs (A) obtained for each sample, the following best-fitting empirical formula was obtained Equation (1):

$$A = S \times ln\,(1/D) + b \quad (1)$$

(A, absorbances; S, slope; D, dilution folds; b, intercept constant)

2) We set the following criteria for a signal to be considered as positive: (i) S <0; (ii) the minimum absorbance of the undiluted sample should be larger than 0.5 (for technical reasons). A plot of all samples' individual $ln(D)$ and (-S) values (for convenience, because the slopes are all <0) with 0.5 absorbance is shown in Figure S2B. The curve-fitting best empirical formula for the plot was obtained as

$$ln(D) = 4.93\,ln\,(X) + 12.84 \quad (2)$$

(X, -1 x slope; D, dilution fold)

3) D<1 meant concentrating the serum which is meaningless. When we set the dilution fold=1 (i.e., y=0), we obtain X=0.074 (slope is -0.074). Accordingly, only samples showing X>0.074 were regarded as autoantibody-positive (Figure S2B).

Supplementary figures

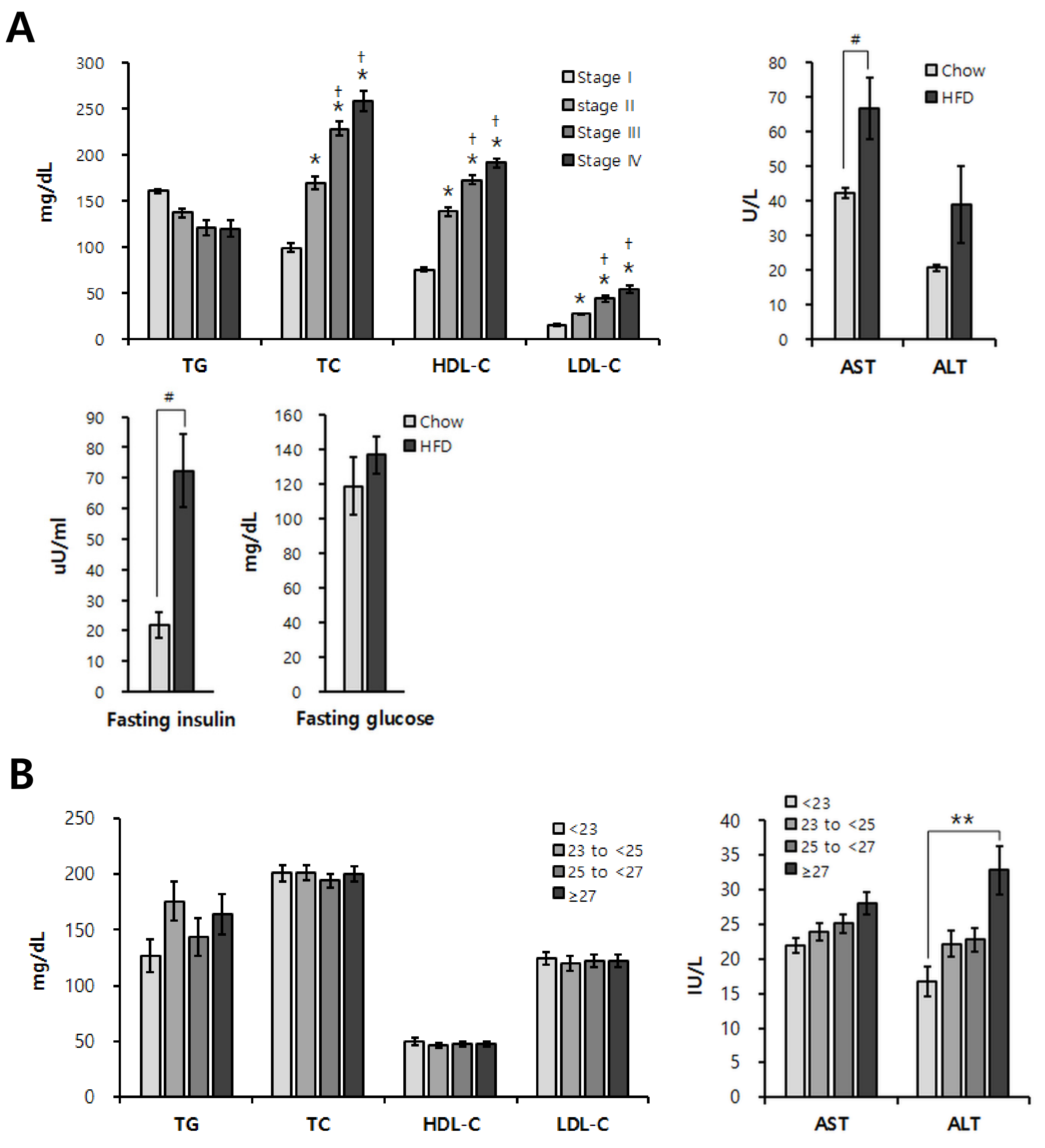

Figure S1. Serum analysis in mouse and human. (A) Serum analysis in mouse. The upper left figure shows the levels of total cholesterol (TC), Triglyceride (TG), high-density lipoprotein (HDL-C) and low-density lipoprotein (LDL-C) in the four stages that are defined in Figure 2B. *, *P*<0.001 compared with Stage I. , †*P*<0.001 compared with Stage I. The upper right figure shows AST and ALT activities in Chow-fed and HFD-fed mouse at 23 weeks of age. The bottom figures show fasting insulin and fasting

glucose levels in Chow-fed and HFD-fed mice at 33 weeks of age. #*P*<0.05. (Chow-fed mice, n=6; HFD-fed mice, n=16). (B) Serum analysis of 107 positive samples according to the BMI group. BMI<23: lean, 23≤BMI<25: overweight, 25≤BMI<27: obese, 27≤BMI: severely obese. n = 23, 26, 27 and 31, respectively. in human. The left figure shows the level of total cholesterol (TC), Triglyceride (TG) high-density lipoprotein (HDL-C) and low-density lipoprotein (LDL-C) according to BMI. #*P*<0.05. Right figure shows AST and ALT activities by BMI. ***P*<0.02. Error bars graphs indicate means±s.e.m. The figure represents three experiments.

A

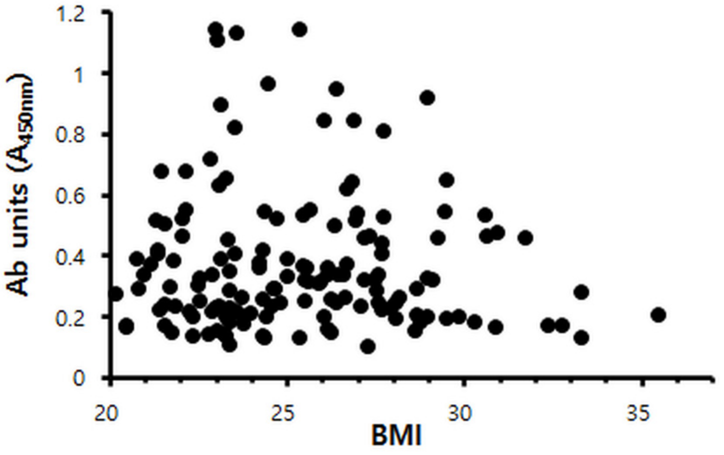

B

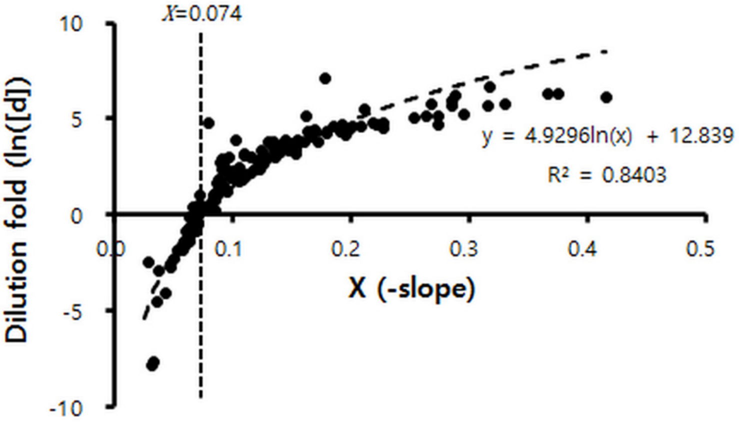

Figure S2. Establishing criteria for true autoantibody positivity. (A) Scatterplot showing the read-outs of th e first ELISA screening of the human serum samples (n=148). Ab (antibody) units indicate the absorbances

of x100 diluted sera at 450nm. (B) Using Equation (2), a standard curve was plotted based on the relations hip between X (negative value of slope) and ln[D] (D, dilution fold) at an absorbance of '0.5' of each seru m sample. Samples showing X>0.074 were regarded as Anti-ApoB100 autoantibody-positive.

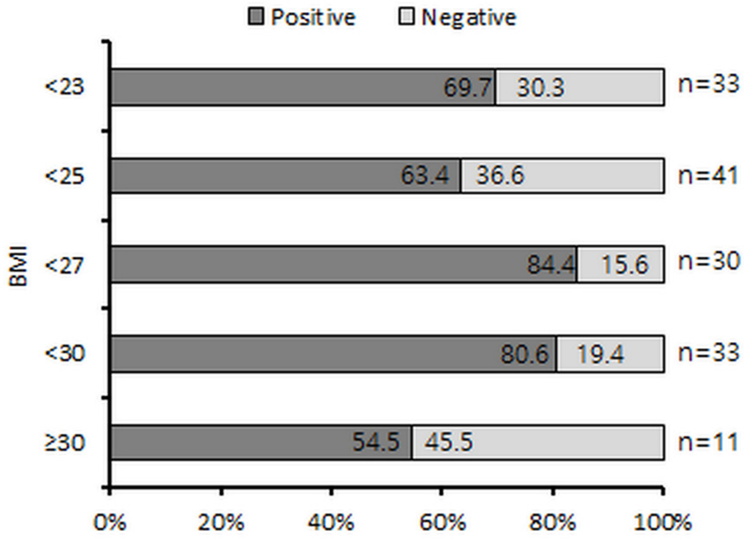

Figure S3. Pattern of autoantibody extinction along obesity progression. The percentage ratios of the Anti-ApoB100 autoantibody positive vs. negative populations also exhibited a biphasic pattern along obesity pro gression, with the most severely obese group also containing the largest negative fraction (45.5%). Human subjects were categorized as "lean" (BMI<23), "overweight" (23≤BMI<25), "mildly obese" (25≤BMI<27), "mo derately obese" or "severely obese" (BMI≥27). n, number of serum samples.
